# The Effect of an Aquatic Exercise Program on Pain and Functional Performance in Overweight Adolescent Runners With Functional Flat Feet

**DOI:** 10.7759/cureus.78444

**Published:** 2025-02-03

**Authors:** Sawani Aphale, Sandeep Shinde, Manoj P Ambali, Makarand Mane

**Affiliations:** 1 Musculoskeletal Sciences, Krishna College of Physiotherapy, Krishna Vishwa Vidyapeeth (Deemed to be University) Karad, Karad, IND; 2 Anatomy, Krishna Vishwa Vidyapeeth (Deemed to be University) Karad, Karad, IND; 3 Medicine, Krishna Vishwa Vidyapeeth (Deemed to be University) Karad, Karad, IND

**Keywords:** aquatic therapy, body mass index, flatfoot, hydrostatic pressure, pain management, postural balance, proprioception, running

## Abstract

Background

Flatfoot is a condition characterized by a diminished or absent medial longitudinal arch, which can lead to pain, altered biomechanics, and reduced functional performance. Overweight adolescents with functional flat feet are particularly at risk due to increased weight-bearing stress, which exacerbates symptoms and impacts daily activities. Aquatic exercise, known for its low-impact nature and supportive environment, has shown promise in alleviating pain and improving functional performance in musculoskeletal conditions. However, its specific effects on functional flat feet in overweight adolescents remain underexplored.

Objective

To evaluate the impact of an aquatic exercise program on pain reduction and functional performance in overweight adolescent runners with functional flat feet.

Methods

This was a comparative study conducted at Krishna College of Physiotherapy, Karad. Ninety-two participants of the age group 13-19 were taken according to inclusion criteria and by using Foot Posture Index-6 and navicular drop test. Participants were divided using a simple random sampling technique and assessed using three outcome measures: the Visual Analog Scale, Clarke’s angle, and the 50-meter sprint run test.

Results

The study interpreted those overweight adolescent runners with functional flat feet exhibited significant improvement in group B than group A. The aquatic exercise program showed extremely significant results (p<0.0001) in the outcomes; Visual Analog Scale, Clarke’s angle, and 50-meter sprint run test than the land-based exercise program.

Conclusion

The study concluded that the aquatic exercise program had a significant impact on the outcomes of Visual Analog Scale, Clarke’s angle, and 50-meter sprint run test. The unique properties of water, including buoyancy and resistance, facilitated enhanced strength, flexibility, and stability while minimizing joint stress. Aquatic exercises led to significantly greater improvements in pain reduction and functional performance compared to land-based exercises in overweight adolescent runners with functional flat feet.

## Introduction

The anatomy of the foot is delineated into three primary regions: the hindfoot, consisting of the talus and calcaneus; the midfoot, comprising the navicular, cuboid, and three cuneiform bones; and the forefoot, composed of the metatarsals and phalanges. In conjunction with the supporting ligamentous structures, these osseous components form three principal arches: the medial longitudinal arch (MLA), the lateral longitudinal arch, and the transverse arch [1].

During the early stages of standing, children typically exhibit a physiological flatfoot accompanied by a valgus position of the hindfoot, which arises because of the inherent flexibility of the joints. As the child matures and the flexibility of the joints decreases, along with subcutaneous fat reduction after three to four years, the MLA begins to develop. This arch is generally fully formed by approximately 10 years of age [2].

Flatfoot, or pes planus, is a condition in which the MLA of the foot collapses, resulting in a postural deformity. Various etiological factors, including traumatic injuries, sustained mechanical stress, obesity, certain systemic illnesses, or abnormal biomechanics can cause this condition. The primary feature of flatfoot is the attenuation or complete flattening of the MLA, which can lead to altered foot structure and function. Flatfoot is defined as a condition in which the MLA descends and flattens under a load of body weight during the propulsive phase of walking [3]. This deformation results from the dynamic forces exerted during ambulation, leading to arch collapse that can affect foot function and biomechanics [4].

Flatfoot is divided into two primary types: flexible flatfoot and rigid flatfoot. In flexible flatfoot, the MLA is visible when the foot is non-weight-bearing, but it flattens when weight is applied. In contrast, a rigid flatfoot is characterized by a complete absence of the arch, regardless of whether the foot is bearing weight. Among these, flexible flatfoot is the most commonly observed form. Flatfoot, particularly when it results in improper foot alignment, can lead to complications in the ankles and knees by disrupting the alignment of the lower limbs. Acquired flatfoot typically arises from excessive force causing arch collapse, compounded by insufficient structural support. The MLA may be underdeveloped in infants and young children due to ligamentous laxity and limited neuromuscular control. In such cases, flatfoot is generally classified as flexible [5].

Marathon runners must maintain peak physical fitness and adhere to a well-balanced diet to optimize their performance during the race. To achieve optimal results, athletes must focus on enhancing cardiovascular endurance, increasing stamina, and refining their ability to efficiently manage and conserve energy throughout the event. However, insufficient fitness levels or excessive physical exertion may elevate the risk of injury among marathon runners [6]. Key factors contributing to such injuries include a history of previous injuries, an overly competitive training approach, and a sudden increase in training intensity or frequency. As running is a weight-bearing activity that involves repetitive impact against gravity, it places considerable strain on the lower extremities, making the knees, ankles, and feet particularly susceptible to injury [7].

Water-based exercise improves balance and coordination by stimulating the visual, vestibular, and proprioceptive systems [8]. The buoyancy of water reduces the impact on joints while providing resistance, which aids in enhancing postural control and body awareness. Aquatic therapy offers the benefit of initiating therapeutic exercises or activities at an earlier stage of the recovery process compared to traditional land-based rehabilitation. This has the potential to accelerate recovery and shorten the overall rehabilitation timeline. Furthermore, it facilitates the targeted enhancement or preservation of neuromuscular and cardiovascular function, all while minimizing the risk of injury due to the buoyancy and resistance properties of water [9].

The properties of water, specifically its density and buoyancy, support the maintenance and early restoration of an optimal walking gait. By diminishing the effects of gravity, water reduces body weight and decreases joint loading, allowing individuals to practice walking with less strain on the musculoskeletal system. This makes it an effective medium for gait training and rehabilitation [9].

Aquatic exercises improve joint mobility, balance, and gait training while promoting flexibility, strength, and endurance. Aquatic therapy plays a key role in functional recovery by facilitating the rehabilitation process and initiating resistance training for runners. It serves both as a recovery modality and as a means to enhance a runner's overall training program, offering a low-impact environment that aids in muscle conditioning and reduces the risk of injury [10,11].

In individuals who are obese or overweight, the impact of increased body mass often outweighs muscle fatigue, as excess weight has been shown to affect plantar pressure distribution and foot posture [12]. These alterations can lead to an elevated risk of foot discomfort and related pathologies, as compared to individuals with normal body weight. The added load on the feet may contribute to abnormal biomechanics, increasing susceptibility to injuries and conditions such as plantar fasciitis or joint misalignments [12,13]. An increase in body weight is linked to higher mid-foot plantar pressure and greater foot-related functional limitations over two years. Additionally, midfoot and heel plantar pressure shifts are associated with changes in foot pain intensity. As body weight and plantar pressure rise, foot pain intensity also increases, with the midfoot being especially susceptible to pressure-induced discomfort due to the increased load on this area [14]. 

The study explores how water-based therapy can address both pain management and performance in a population typically underserved in existing research. The focus on aquatic exercise which includes the use of water properties offers unique insights into rehabilitation for functional flat feet in young athletes. So the study was conducted to evaluate the impact of aquatic exercise on pain and functional performance in overweight adolescent runners with functional flat feet.

## Materials and methods

Selection of subjects

The study sample consisted of 92 recreational adolescent runners from Karad, aged between 13 and 19 years, who regularly engaged in exercise and were capable of running long distances. All participants voluntarily consented to participate in the study by providing written informed consent. Out of 92 participants, four participants were lost to follow-up due to various reasons. It was a single-blinded study where the participants do not know the treatment they received. Random allocation was done by the simple random sampling method with the help of SPSS 26.0 software (IBM Corp., Armonk, NY, USA). The runners were randomly assigned to either the experimental group (n=44) or the control group (n=44). Before participation, the participants were informed about the study protocol, their rights, and any potential risks. This study was conducted on humans and was approved by the Institutional Ethics Committee of Krishna Vishwa Vidyapeeth, “Deemed to be University,” Karad. The sample size was 92 which was calculated by 4pq/L^2^, where p-prevalence, q-100-p, L- allowable error. Inclusion criteria were participants with a BMI ranging from 25 to 29.9 kg/m², runners who engage in a daily routine of running at least three km thrice a week for one year, individuals with a history of pain associated with running for more than six months, individuals with a navicular drop between 10-15 mm in both feet, exhibiting non-neutral foot posture, participants were diagnosed with bilateral functional flatfoot, based on the navicular drop test and a Foot Posture Index-6 (FPI-6) score between +5 and +12. Exclusion criteria of the study were individuals with open wounds, skin conditions, or fever, participants with aquaphobia, individuals with other foot deformities, lower limb fractures, postoperative cases, or a history of trauma.

Assessment for flatfoot

Sit-to-Stand Navicular Drop Test

The assessment was initiated by locating and marking the navicular tuberosity on both feet of a barefoot, seated participant using a hypoallergenic black marker. The neutral position of the subtalar joint was identified through palpation. A rigid, blank paper card was placed medially on the foot, and the navicular tuberosity was marked using a ruler. The navicular height (NH) was measured in standing, with one foot positioned on a medical scale and the contralateral foot placed on a support of equivalent height to the scale platform. The participant was instructed to shift 80% of their body weight onto the foot being measured. The navicular height was then recorded on the card. This was calculated as the difference between the navicular height in the seated position and the standing position [15]. The results of the SSNDT were categorized as follows: supinated: <5 mm, neutral: 5-9 mm, pronated: 10-15 mm. According to SSNDT results, participants were divided into three categories: both feet neutral, one foot neutral, and the other non-neutral (either pronated or supinated), both feet non-neutral.

Foot Posture Index-6

The FPI-6 is used to assess foot posture by evaluating six anatomical criteria, each scored from 0 (neutral) to +1 or +2 (pronated) and -1 or -2 (supinated). The evaluation begins with palpating the talar head on both the medial and lateral sides of the ankle to assess its position. The curvature of the supra and infra-lateral malleolus is then observed to detect any changes in curvature above and below the lateral malleolus. The calcaneal frontal plane position is examined by observing the alignment of the calcaneus, using the orientation of the calcaneal tendon as a reference. The bulging of the talonavicular joint is assessed for any prominence or abnormal protrusion. The height and alignment of the medial longitudinal arch are checked for congruency and any deviations from neutral. Finally, the degree of forefoot abduction or adduction is measured to evaluate the alignment of the forefoot. This thorough assessment provides a comprehensive understanding of foot posture, helping to identify whether the individual exhibits pronated or supinated characteristics [16].

**Figure 1:**
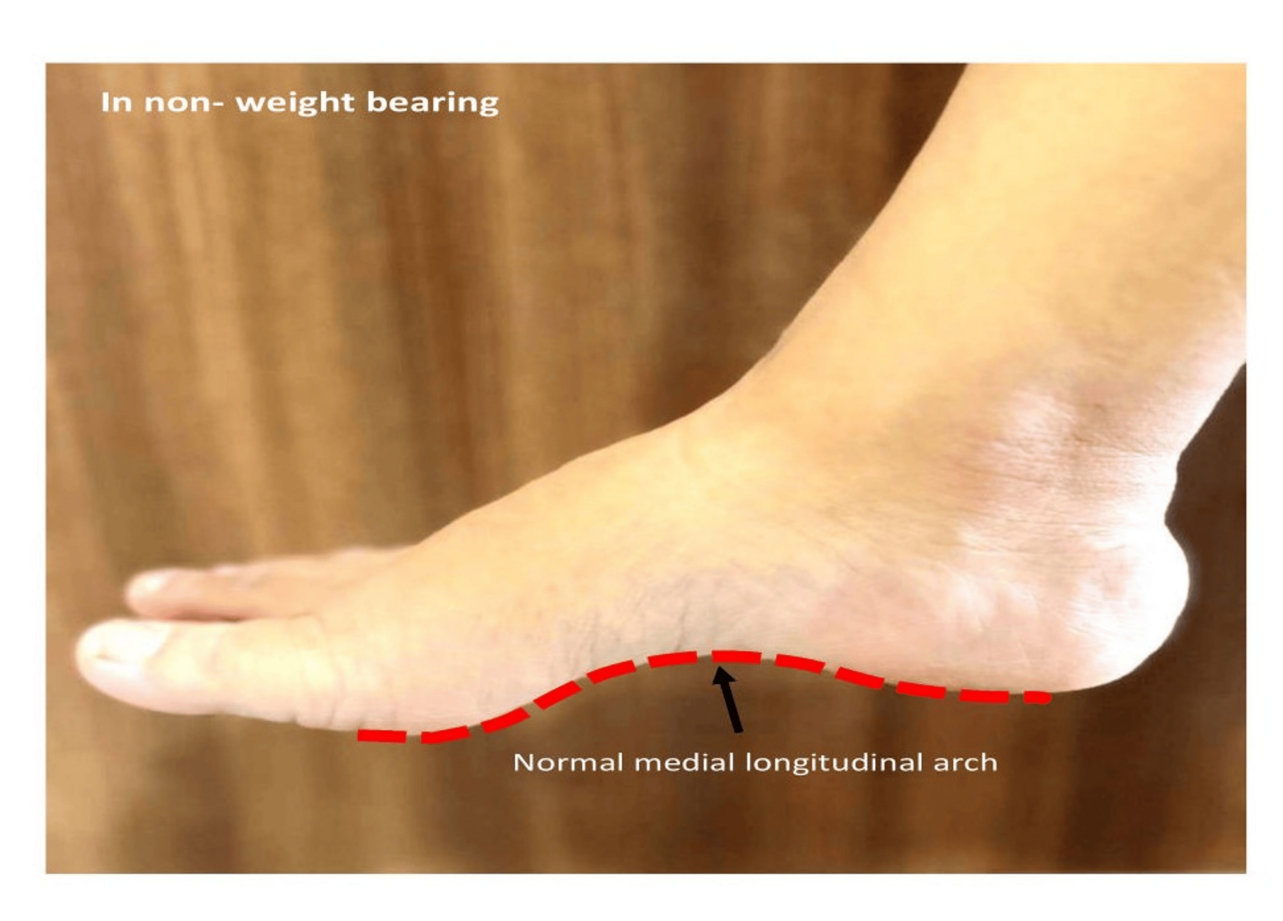
Normal medial longitudinal arch in non-weight bearing position

In Figure 1, the medial view showcases the normal medial longitudinal arch of the right foot, emphasizing its role in weight distribution and shock absorption.

**Figure 2:**
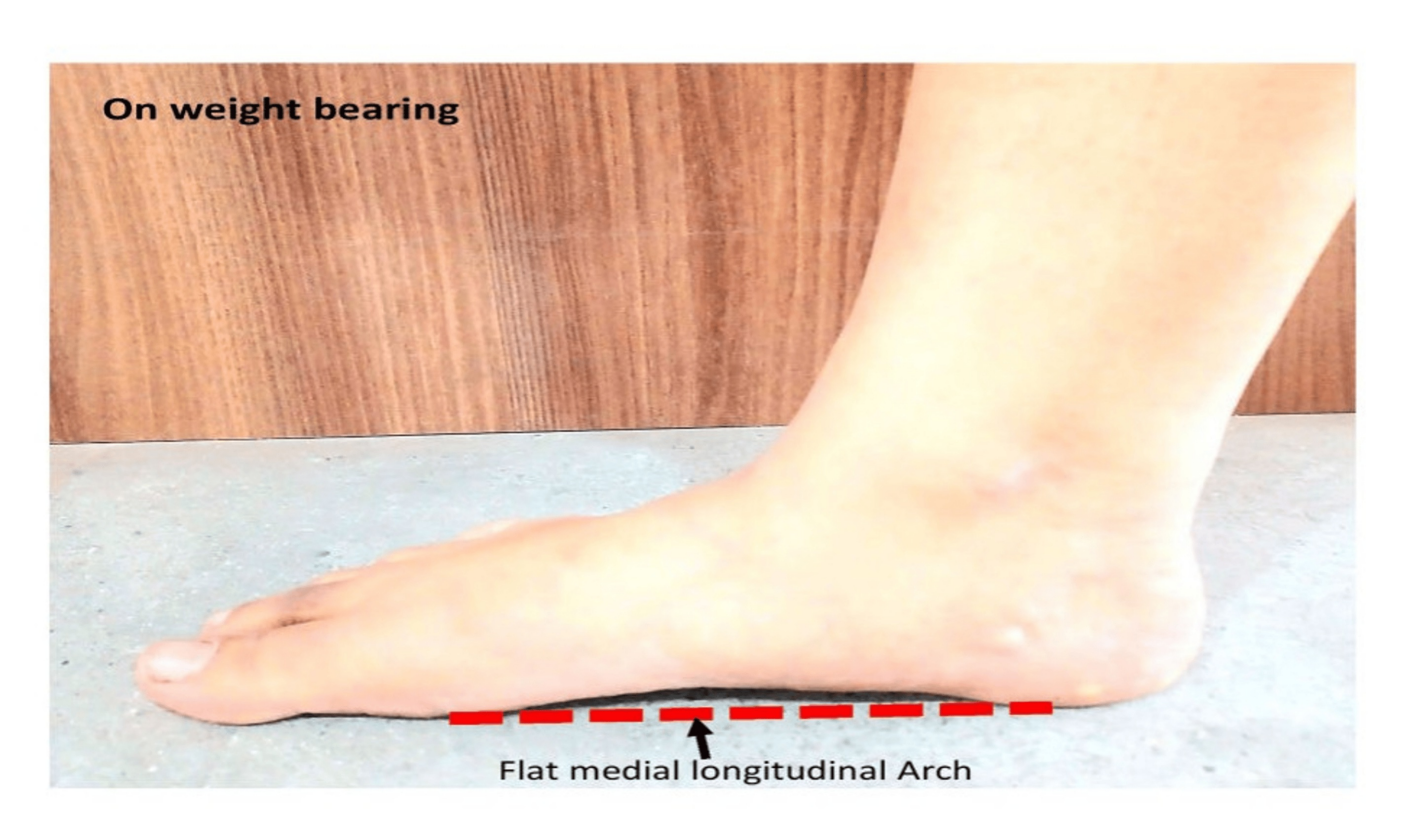
Flat medial longitudinal arch on weight bearing

In Figure 2, the medial view showcases the flat medial longitudinal arch of the right foot on weight bearing (functional flatfoot).

Treatment protocol

Treatment includes three phases, warm-up, active exercise training, and cool-down phase. Treatment was performed five times a week and progression was shown from 0 to six weeks in both groups. 

**Table 1:** Treatment protocol for group A (land based) and B (aquatic exercises) from 0 to six weeks

GROUP A (Land based)		GROUP B (Aquatic)	
Exercise program	Repetitions/ Duration	Exercise program	Repetitions/ Duration
Warm up		Warm up	
Deep breathing exercises including pursed lip breathing, thoracic expansion exercises	3 mins	Deep breathing exercises including pursed lip breathing, thoracic expansion exercises	3 mins
Whole body activation exercises		Whole body activation exercises	
Wall push ups	5 sets – 5 sec hold	Water push ups	5 sets – 5 sec hold
Arm circles	10 times	Underwater arm circles	10 times
Planks	5 times – 5 sec hold	Water planks	5 times – 5 sec hold
Sit ups	5 times	Cross country ski	5 times
Torso twists	5 times	Torso twists	5 times
Toe curls	10 times	Toe curls	10 times
Walking forward	3 mins	Walking forward	3 mins
Walking backward	3 mins	Walking backward	3 mins
Walking sideways	3 mins	Walking sideways	3 mins
Toe curls	10 times	Toe curls	10 times
Exercise training (0-3 weeks)		Exercise training (0-3 weeks)	
Ankle toe movements	10 times	Marching on spot	10 times
Rolling – tennis ball under foot	10 times	Tandem stance	3 sets
Bilateral heel raises	5 times – 5 sec. hold	Step up and step down	10 times
Towel curls	10 times	Single leg stance with while catching and throwing a ball	5 times – 2 sets
Isometrics – hamstrings and quadriceps	5 times – 10 sec, hold	Single leg stance with leg swing with eyes open and closed	5 times – 2 sets
Three way kicks with the support of chair	5 times – 2 sets	Single leg stance with eyes open and then eyes closed.	5 times- 2 sets
Short foot exercise	10 times	Double leg calf raise	3 times – hold 5 sec
Step up and down	10 times	Short foot exercise	10 times
Tandem stance	3 sets	Deep water walking (Walking where sand is present in water.	5 mins
Exercise training ( 4-6 weeks )		Exercise training (4-6 weeks)	
Sit kicks	10 times	Resistance Flutter kicks	10 times
Jumping jacks	10 times	Jumping jacks	10 times
Partial squats	10 times	Sit kicks in deep water	10 times
Lunges	10 times	Water jog	3 mins
Double leg calf raise	10 times -5 sec. hold	Double leg calf raise	5 times – 10 sec hold
Resistance flutter kicks	10 times	Resistance flutter kicks	10 times
Jogging	5 mins	Lunges	10 times
Kicking the ball in different directions	10 times	Side to side alternate leg bounds	10 times
Single leg stance with eyes open and then eyes closed	10 sec hold – 5 sets	Single leg stance with eyes open and then eyes closed.	10 sec hold – 5 sets
Tandem stance	10 sec hold – 5 sets	Pool tuck jumps	10 times
Ankle dorsiflexion- plantarflexion with the help of resistance band	10 times – 2 sets	Deep water walking (Walking where sand is present in water.)	10 mins
Cool down		Cool down	
Static stretching of Tendo Achilles and plantar fascia	10 sec hold – 3 sets	Static stretching of Tendo Achilles and plantar fascia	10 sec hold – 3 sets
Toe walking	3 sets	Toe walking	3 sets
Heel walking	3 sets	Heel walking	3 sets
Relaxation techniques	5 mins	Relaxation techniques	5 mins

Exercise intensity was monitored by waterproof fitness monitoring wearable devices which track the heart rate, oxygen saturation, and exact exercise duration before and after the exercise session. Moderate-intensity exercises were given to both groups including warm-up, active phase of exercise training, and cool-down phase. Also to work-rest ratio was maintained properly during both groups' exercises.

Selection of outcome measures

Visual Analog Scale

The VAS was used to measure pain intensity. This scale ranges from 0 to 10, where 0 represents "no pain" and 10 indicates the "worst pain imaginable." The VAS is a one-dimensional tool commonly used to assess pain intensity and is applicable across a wide range of age groups. It provides a simple yet effective means of evaluating subjective pain experiences [17].

50-Meter Sprint Test

The 50-meter sprint test was taken to assess the sprinting speed of athletes. Sprint times were manually recorded to the nearest hundredth of a second using a stopwatch. The required equipment for the test included a measuring tape, a 50-meter marked grassy field, a starting clapper, a skilled timer, and a scorekeeper. Each participant completed a single 50-meter sprint, and their time was recorded in seconds. Participants were allowed up to two practice runs to familiarize themselves with the test, with encouragement to exert maximum effort during the sprints. The test began with a standing start, and a maximum of two trials were conducted, with the fastest time from these trials used for analysis [18].

Clarke’s Angle

Clarke’s angle (CA) was measured using a static footprint. To obtain the footprint, each participant was instructed to step onto a flat pad with water-soluble ink, pressing firmly with both feet. They were then asked to step forward, placing their right foot onto a graph sheet, followed by the left foot. The participant was required to stand in a relaxed stance with equal weight on both feet, looking forward for two seconds before stepping forward to clear the graph sheet. To calculate CA, a marker pen, ruler, and protractor marked at one-degree intervals were used. CA was determined by measuring the angle between two lines: the first, a medial tangential line connecting the medial edges of the first metatarsal head and the heel, and the second, a line connecting the first metatarsal head and the concavity of the MLA. Clarke’s angle was classified as follows: “normal” (CA 42°-54°), “mild flatfoot” (CA 35°-41°), “moderate flatfoot” (CA 30°-34.9°), “severe flatfoot” (CA <30°), and “high arched foot” (CA >54°) [19].

Statistical analysis

The statistical analysis of the obtained data was conducted using IBM SPSS statistics software version 26. A paired t-test was employed to assess differences in outcome parameters before and following the intervention. An unpaired t-test was used to compare the outcome measures between the two groups. The arithmetic mean and standard deviation were calculated for each outcome variable. The arithmetic mean was determined by summing all individual values and dividing by the total number of observations.

## Results

Table 2 demonstrated about demographic variables of the participants consisting of age, gender, and BMI correlated with the patients. The study includes a total of 88 (100%) participants aged 13-19 years, from which 44 (50%) were included in each age group and then subdivided into two groups of 22 (25%) participants in each group. The study includes both 44 (50%) males and 44 (50%) females. According to BMI, patients categorized in the overweight category 25-29.9 kg/m2 were included in both groups.

**Table 2:** Sociodemographic status of participants BMI-Body Mass Index

Demographic variables		Total no. of participants	Control group	Experimental group
Age	13-16 years	44 (50%)	22 (25%)	22 (25%)
	17-19 years	44 (50%)	22 (25%)	22 (25%)
BMI	25-29.9 kg/m^2^	88 (100%)	44 (50%)	44 (50%)
Gender	Males	44 (50%)	20 (22.72%)	24 (27.27%)
	Females	44 (50%)	24 (27.27%)	20 (22.72%)

Table 3 states pain assessment by VAS. In the 13-16 yrs age group, both the groups had significant improvement in pain at rest and on activity. In the 17-19 yrs age group, group B had significant improvement p<0.0001 than group A, both at rest (p=0.0008) and on activity (p=0.0006). The mean difference between group B for pain reduction was greater than group A in both groups. In the table, t values are included.

**Table 3:** Pain assessment by visual analog scale VAS-Visual Analog Scale

Study Groups	Age group	VAS assessment	Pre values	Post values	P value	t value
Group A	13-16 years	At rest	1.54±0.5096	0.68±0.7162	0.0002	4.557
		On activity	3.45±0.5958	2.22±0.6853	<0.0001	7.085
	17-19 years	At rest	1.59±0.5032	1.09±0.5264	0.0008	3.924
		On activity	3.54±0.5096	2.72±0.9351	0.0006	4.006
Group B	13-16 years	At rest	1.63±0.4924	0.09±0.2942	<0.0001	14.223
		On activity	3.95±0.5755	1.13±0.5602	<0.0001	18.042
	17-19 years	At rest	1.42±0.5071	0.38±0.4946	<0.0001	8.143
		On activity	3.95±0.5755	2.18±0.7327	<0.0001	8.549

Table 4 states Clarke’s angle assessment for flat feet. According to the results given, it was interpreted that, in the age group of 13-16 years, group B had a significant improvement in the angle p<0.0001 than group A in both feet. In the other age group also, group B had significant improvement than group A. Comparing the values of pre and post-interventions of group B in both the age groups, it was seen that the 13-16-year-old age group had a greater mean difference in improving the angle than another age group. In the table, t values are included.

**Table 4:** Clarke's angle for assessment of flat feet

Study groups	Age group	Side of the foot	Pre values	Post values	P value	t value
Group A	13-16 years	Left foot	36.45±0.5958	36.81±0.9069	0.0079	2.935
		Right foot	36.31±0.6463	36.86±0.7743	0.0023	3.464
	17-19 years	Left foot	37.18±1.053	37.50±1.144	0.0051	3.130
		Right foot	36.45±0.5096	36.59±0.5032	0.0829	1.821
Group B	13-16 years	Left foot	38.59±1.532	42.09±1.019	<0.0001	9.593
		Right foot	38.81±1.368	42.04±1.463	<0.0001	8.680
	17-19 years	Left foot	39.00±1.378	40.57±1.630	<0.0001	8.883
		Right foot	39.80±1.209	41.09±0.8891	<0.0001	5.139

Table 5 evaluated the pre- and post-test assessments in the 50-meter sprint run test. In the 13-16 years of age group, group B had an extremely significant improvement p<0.0001 to group A (p=0.0157) and in the 17-19 years of age group, group B had a significant improvement (p=0.0045) to group A (p=0.0829). In the table, t values are included.

**Table 5:** 50-meter sprint run test assessment

Study groups	Age group	Pre values	Post values	P value	t value
Group A	13-16 years	6.32±0.1378	6.25±0.1403	0.0157	2.628
	17-19 years	6.17±0.0643	6.15±0.0597	0.0829	0.0829
Group B	13-16 years	6.28±0.1612	6.00±0.1090	<0.0001	6.803
	17-19 years	6.14±0.081	6.04±0.1248	0.0045	3.202

Table 6 states the post-test assessment values between the groups. In the age group 13-16 years, for pain at rest p=0.0009, pain on activity p<0.0001. In the 17-19 years of age group, for pain at rest, p<0.0001, and on activity p=0.0014 was seen. While analyzing Clarke’s angle for both age groups, p<0.0001 was obtained by unpaired t-test. For the 50-meter sprint run test, p<0.0001 was obtained for the age group of 13-16 years and p=0.0002 was obtained for 17-19 years participants. In the table, t values are included. As per the analysis, a significant difference was observed between the post-assessment values of the two groups.

**Table 6:** Between the group analysis for all outcome measures VAS-Visual Analog Scale

Outcome measures	Age groups	Post-assessment values (control group)	Post-assessment values (experimental group)	p value	t value
VAS (At rest)	13-16 years	0.68±0.7162	0.09±0.2942	0.0009	5.054
VAS (On activity)		2.22±0.6853	1.13±0.5602	<0.0001	8.168
VAS (At rest)	17-19 years	1.09±0.5264	0.38±0.4946	<0.0001	0.710
VAS (On activity)		2.72±0.9351	2.18±0.7327	0.0014	3.0152
Clarke’s angle	13-16 years (Left foot)	36.81±0.9069	42.09±1.019	<0.0001	27.366
	13-16 years (Right foot)	36.86±0.7743	42.04±1.463	<0.0001	20.758
	17-19 years (Left foot)	37.50±1.144	40.57±1.630	<0.0001	10.226
	17-19 years (Right foot)	36.59±0.5032	41.09±0.8891	<0.0001	29.217
50-meter sprint run test	13-16 years	6.25±0.1403	6.00±0.1090	<0.0001	9.333
	17-19 years	6.15±0.0597	6.04±0.1248	0.0002	5.274

## Discussion

The objective of this study was to evaluate the impact of a six-week aquatic training program on pain and functional performance in recreational runners with functional flatfoot. The study involved 88 participants who ran 3 km per day and fell into the overweight BMI category. The results indicated that Group B demonstrated a highly significant improvement in all outcomes compared to Group A. Additionally, runners between the ages of 13 and 16 demonstrated more notable improvements compared to those in the 17-19 age group. These findings underscore the potential benefits of aquatic training and deep-water walking for individuals with functional flatfoot, highlighting their positive impact on pain reduction and functional performance.

One previously reported case involving a 17-year-old female athlete diagnosed with Achilles tendinopathy in the left foot experienced point tenderness and swelling over the Achilles tendon, specifically in the middle section proximal to the calcaneal insertion. The tendon was painful upon waking in the morning and felt stiff when she began running. aquatic rehabilitative exercises for Achilles tendinopathy offer valuable insights into athlete training. The unique properties of water, particularly in a gravity-reduced environment, significantly accelerate recovery, providing advantages over traditional land-based training methods [20].

Another study highlighted the significance of aquatic-based exercise training for elite athletes. Aquatic rehabilitation has the potential to expedite the recovery process after injury by enabling effective cardiovascular and musculoskeletal training through water-based exercises. The buoyancy and resistance properties of water provide a low-impact environment for rehabilitation, allowing individuals to perform exercises that may be challenging or too strenuous on land. The pool can be used not only during the rehabilitation phase but also in the post-recovery period, serving as an adjunctive tool to further enhance recovery and preserve physical conditioning [21].

The previous study suggests that adjusting various constraints during aquatic training offers athletes a distinctive opportunity to maintain or improve their physical capacities without subjecting the musculoskeletal system to the high loading and stress typically encountered in land-based training. While highly trained athletes may require more specific training and progressive overload, short-term or intermittent aquatic training can serve as an effective complement to land-based programs. This is especially beneficial during general physical preparation phases, transitional periods (such as return-to-training or return-to-play after extended breaks), preseason training camps, and competitive seasons. Aquatic-based rehabilitation can significantly support the athletic population in enhancing their performance [22].

A previous study highlighted the positive effects of aquatic training on male long-distance runners. The study involved 10 male runners, who were randomly divided into two groups. The aquatic training regimen consisted of water walking, jogging, and jumping after downhill running over three consecutive days. Muscle soreness was assessed in both groups, with the aquatic training group showing a significant reduction in muscle soreness compared to the control group [23].

Another study involved 100 recreational male and female runners, aged 25 to 35 years, who met the specified inclusion and exclusion criteria. The participants were randomly assigned to two equal groups. Over 6 weeks, the aquatic training program incorporated aerobic, resistance, and endurance training components. Aquatic therapy was found to be effective in reducing pain, improving range of motion, and enhancing balance and coordination. Additionally, it provided a soothing and relaxing effect for participants after treatment, helping to alleviate muscle fatigue and reduce the overall workload on muscles. The study concluded that pre-rehabilitation aquatic training plays a crucial role in enhancing a runner's performance in the future [8].

The present study emphasizes the impact of aquatic training for recreational runners with functional flatfoot, demonstrating its positive effect on both pain reduction and functional performance.

Limitations

The study was performed at a single location, which may constrain the applicability of the results to other populations or settings. As the study duration was six weeks, it was not possible to take long-term follow-up of these individuals. Conducting a long-duration study or a study that will involve long-term follow-ups will be considered for future scope. Participants in the aquatic therapy group may have been more motivated due to the novelty and perceived enjoyment of the exercise, leading to a potential Hawthorne effect.

Strengths

This study explored the use of aquatic exercise for overweight adolescent runners with functional flat feet, an area that has not been extensively studied. It provides valuable insights into the potential of water-based rehabilitation as an alternative method for improving foot health and overall performance.

Future recommendations

To improve the generalizability of the results, future research should involve a more diverse range of populations, and demographics, encompassing adolescents of varying body compositions, different severities of flat feet, and individuals from diverse geographic locations. This will allow for a more comprehensive understanding of the effectiveness of aquatic training across various populations.

## Conclusions

The study concluded that an aquatic exercise program significantly improved key outcomes, including pain reduction, Clarke's angle, and performance on the 50-meter sprint test. Notably, runners aged 13-16 years showed more significant improvements than those in the older age group, emphasizing the importance of early rehabilitation for adolescents with functional flatfoot to prevent future complications. The unique physical properties of water, such as buoyancy and resistance, played a crucial role in enhancing strength, flexibility, and stability while minimizing joint stress. These benefits make aquatic therapy particularly advantageous for individuals with musculoskeletal conditions, such as overweight adolescent runners with functional flat feet, as it supports effective rehabilitation and conditioning without worsening existing issues. When compared to land-based exercises, aquatic exercises led to more substantial improvements in both pain management and functional performance, resulting in faster physical recovery and enhanced performance. Therefore, aquatic therapy can be considered a valuable alternative or complement to traditional land-based exercise programs, especially for individuals with specific biomechanical challenges. The findings underscore the potential of aquatic therapy as a safe, effective, and low-impact approach to improving physical health and athletic performance.
